# Art in Time and Space: Context Modulates the Relation between Art Experience and Viewing Time

**DOI:** 10.1371/journal.pone.0099019

**Published:** 2014-06-03

**Authors:** David Brieber, Marcos Nadal, Helmut Leder, Raphael Rosenberg

**Affiliations:** 1 University of Vienna, Department of Basic Psychological Research and Research Methods, Faculty of Psychology, Vienna, Austria; 2 University of Vienna, Department of Art History, Faculty of Historical and Cultural Studies, Vienna, Austria; CSIC-Univ Miguel Hernandez, Spain

## Abstract

The experience of art emerges from the interaction of various cognitive and affective processes. The unfolding of these processes in time and their relation with viewing behavior, however, is still poorly understood. Here we examined the effect of context on the relation between the experience of art and viewing time, the most basic indicator of viewing behavior. Two groups of participants viewed an art exhibition in one of two contexts: one in the museum, the other in the laboratory. In both cases viewing time was recorded with a mobile eye tracking system. After freely viewing the exhibition, participants rated each artwork on liking, interest, understanding, and ambiguity scales. Our results show that participants in the museum context liked artworks more, found them more interesting, and viewed them longer than those in the laboratory. Analyses with mixed effects models revealed that aesthetic appreciation (compounding liking and interest), understanding, and ambiguity predicted viewing time for artworks and for their corresponding labels. The effect of aesthetic appreciation and ambiguity on viewing time was modulated by context: Whereas art appreciation tended to predict viewing time better in the laboratory than in museum context, the relation between ambiguity and viewing time was positive in the museum and negative in the laboratory context. Our results suggest that art museums foster an enduring and focused aesthetic experience and demonstrate that context modulates the relation between art experience and viewing behavior.

## Introduction

Visual exploration is an active and dynamic process of gathering information about the world. Contextual and personal factors influence this dynamic visual exploration [1], [2]. On the one hand, context can facilitate or hinder the recognition and identification of objects [3]; on the other, motivational, emotional, and cognitive states influence where people look and for how long [4], [5]. Thus, the time it takes to visually explore an object can inform about its emotional relevance, interestingness, or even its aesthetic appeal. So far, in the domain of art little attention has been paid to the interrelations among duration of visual exploration, subjective experience, and context. This is surprising, considering that art experience is a temporally evolving one, that it involves a large variety of psychological processes, and that it is tightly linked to specific contexts, such as art museums. The main objective of this paper is, therefore, to examine the relations among viewing time, art experience, and context.

### Viewing time and the experience of images and art

Several studies have demonstrated that certain stimulus features and subjective experiences condition viewing time (see [6] for an early bibliography). For example, greater stimulus size, complexity, or novelty, lead to longer viewing times for abstract patterns, line drawings, or pictures of real-world scenes [7]–[11]. With regard to subjective experience, stimuli evaluated as interesting or emotionally arousing receive longer looks [12], [13]. Interestingly, also aesthetic features such as facial attractiveness have a positive relationship with viewing time [14], [15]. However, what is known about the relation between viewing time and the experience of art?

Although empirical aesthetics is the second oldest branch of experimental psychology [16], little is known about how the experience of art unfolds over time. The experience of art is a complex one, as it emerges from an intricate interaction among processes of perception, attention, memory, decision-making, affect, and emotion [17]. Even the experience of the static arts “is always a temporally moving process of doing and undergoing where experience is developed cumulatively and brought to fulfillment” [18], p. 26. How long does it take for aesthetic experience to develop? What is the relation between the quality and the duration of aesthetic experience? Do contextual features modulate this relation? These fundamental questions remain largely unanswered.

Only few studies have examined the relation between aesthetic evaluation and viewing time with regard to the perception of art. Research in art museums showed that 1) the number of artworks in an exhibition room affects viewing time [19], 2) viewing time commonly decreases over the course of the exhibition - a phenomena called *museum fatigue* (for an overview see [20]), and 3) the average time spent on viewing artworks is generally short. Smith and Smith [21] recorded how long people viewed six of the paintings in the permanent collection of The Metropolitan Museum of Art (containing over a million objects). They reported a median viewing time of 17 seconds (mean of 27 seconds). Around 50 percent of the looks lasted less than 10 seconds, one quarter to one third of the looks lasted around half a minute, and only 10 percent of the looks lasted longer than a minute. Moreover, mean viewing time significantly differed among artworks and participants, suggesting that both artwork-specific features and personal characteristics affect viewing time.

However, Smith and Smith [21] performed their measurements using manual chronometry, and they did not collect data about people's subjective experience of each artwork. Heidenreich and Turano's [22] study aimed to overcome these limitations. They analyzed the correlation between viewing time and art experience, and they measured viewing time and eye movement patterns with a head mounted eye tracker while participants (solely) viewed 15 selected artworks exhibited in a museum. Participants were asked to rate each artwork on pleasantness, interestingness, beauty, power, clarity, and orderliness. Similar to Smith and Smith, they found large individual differences with viewing times ranging from 20 to 82 seconds. Surprisingly, however, viewing time did not significantly correlate with any of the art experience scales. This would imply that, in contrast to other types of visual stimuli, viewing time for artworks is not related to subjective experience. However, due to the small sample size (N = 4) the generalizability of these results is limited and has to be taken with caution. Nevertheless, these museum studies suggest that average viewing time for artworks is around 20 seconds with large variations among people and artworks. The relation between viewing time and the experience of art, however, remains unclear.

### Context and the experience of art

Art is always experienced in a specific context. The semantic context created by providing certain information, such as artwork title [23], [24], art historical facts [25], or about authenticity [26]–[28], has appreciable effects on people's response to and evaluation of art, as well as on the underlying neural processes [29]–[31].

In contrast to these semantic context effects, only few studies have investigated the effect of physical context. On a local scale, the near physical context are other surrounding artworks which can lead to contrast and assimilation effects [32]. Physical context on a larger scale includes the space in which artworks are presented, typically museums, galleries, or exhibition rooms of private collectors with variable interior designs (big or small rooms with white or rather colorful walls). This physical context is important for the classification of an object as a work of art. Moreover, all subsequent cognitive and emotional processes involved in art perception are always embedded in a context [17]. Thus, different physical contexts might lead to differences in aesthetic processing. Psychological research on the perception of art, however, has mostly been conducted in the laboratory context. Experiments in laboratories enable researchers to investigate specific factors while controlling for confounding variables. This laboratory approach has increased our knowledge about the psychological mechanisms involved in the experience of art. But, to what extent are these results generalizable to real world situations? How does the experience of art in the laboratory and the museum differ?

Locher, Smith and Smith [33] showed that pictorial features such as symmetry or complexity, were evaluated in a similar way when viewing the original artworks in the museum and their reproductions on the computer screen in the laboratory. There seemed to be, however, differences in the hedonic value between the museum and laboratory settings. People found original artworks in the museum as more interesting and pleasing than their reproductions in the laboratory. These results were confirmed and extended by a study directly comparing art experience in the laboratory and the museum [34]. People in the museum liked the artworks more, they found them more interesting, and their affective response was more positive and arousing than compared to the laboratory context. These findings suggest that there is an enhanced art experience in the museum.

### The present study

The aim of this study was to determine whether the context in which artworks are experienced (museum or laboratory) influences the time participants choose to view them and, especially, whether it influences the relation between the experience of art and viewing time. We use the term context broadly to refer to a specific setting characterized by its typical features such as room size, lighting, and type of objects (e.g. original artworks vs. reproductions). We expected viewing time for artworks and corresponding labels to be predicted by beholders' subjective experiences, artwork related features, and contextual factors. Assuming that the museum context fosters a focused art reception, our hypothesis was that average viewing time for artworks would be longer in the museum than in the laboratory. Although this may seem obvious, it has actually not been empirically tested before. We also expected to replicate previous studies [34], and find an enhanced art experience in the museum. Finally, we hypothesized that context would modulate the relation between viewing time and art experience. Accordingly, we measured viewing time for artworks and labels with a mobile eye tracker while people freely viewed an art exhibition either in the museum or in the laboratory.

## Methods

### Ethics Statement

This study was conducted in accordance with the Declaration of Helsinki (revised 1983) and guidelines of the Faculty of Psychology, University of Vienna. According to the Austrian Universities Act 2002 (UG2002) which held at the time the study was carried out, only medical universities were required to appoint ethics committees for clinical tests, application of medical methods, and applied medical research. Therefore, no ethical approval was required for the present study. All participants provided written informed consent prior to their participation and could withdraw at any time during the study without further consequences.

### Participants

Forty-four psychology students from the University of Vienna participated in this study. Half of the participants were randomly assigned to the museum group (*n*_MG_ = 22), the other half to the laboratory group (*n*_LG_ = 22). Participant's age ranged between 18 and 31 years (*M*_MG_ = 23; *M*_LG_ = 24) and both groups included similar number of men and women (men: *n*_MG_ = 6, *n*_LG_ = 8; women: *n*_MG_ = 16, *n*_LG_ = 14). All participants had normal or corrected vision, no formal training in arts or art history and received course credit for taking part.

### Stimuli

All stimuli were taken from an original art exhibition held in a museum of contemporary art in Vienna (www.musa.at). The exhibition was called “distURBANces. How fiction beats reality.” and contained 14 art photographs or series of related art photographs from the same artist. The photographs depicted humans and objects in blends of urban and natural environments, staged, composed, or manipulated by the artists. We chose this particular exhibition of art photographs because it provided the opportunity for a greater similarity between originals and reproductions than exhibitions of paintings, installations, or sculptures. While the museum group saw the original artworks in the museum, the laboratory group viewed high-resolution digital reproductions of the original artworks presented on a 31” computer screen (2560×1600 pixels). For both groups, each artwork or series of artworks from the same artist was accompanied with a label giving information about the artwork, the artist, and the artist's work in general. During the free viewing block in the laboratory group, we used the online presentation software Prezi (www.prezi.com) for stimulus presentation. This allowed participants to zoom in to the screen and to go backwards and forwards through the order of artworks by imitating movement from one artwork to the next. Order of artworks and size relations between adjacent artworks were the same as in the museum context. For the rating block, artworks were presented with E-Prime 2.0 software (Psychology Software Tools, Pittsburgh, PA).

### Apparatus

The time participants in both groups looked at the artworks and labels was measured binocularly with the mobile Eye Tracking Glasses from SensoMotoric Instruments (SMI; Teltow, Germany). Data was stored with a sample rate of 30 Hz on a mobile recording unit.

### Procedure

The study was divided into a free viewing block, a rating block, and a questionnaire block. In the first block, participants in both groups, one by one, freely viewed the artworks in the exhibition. The museum group viewed the original artworks in the museum; the laboratory group viewed digital reproductions of these artworks on the screen in the laboratory. We emphasized that they could spend as much time as they wanted looking at the exhibition. Before they entered the exhibition hall, the museum group received a map of the exhibition room with a predefined walking path. This ensured that both groups saw the artworks in the same order while keeping the curatorial arrangement of artworks intact. It was important that both groups had a similar amount of options in this free viewing block. Therefore, just as the museum group, participants in the laboratory group were able to go backwards and forwards in the presentation order. Moreover, to view an artwork in more detail or to read the accompanied text information it was possible to zoom in and out. Eye movements were recorded all through the exhibition.

In the rating block, after the participants had freely viewed all artworks and eye movements had been recorded, participants were asked to rate liking (“*How much do you like this artwork?*”), interest (“*How interesting do you find this artwork?*”), understanding (“*How much do you have a sense of understanding this artwork?*”), and ambiguity (“*How ambiguous is this artwork for you?*”) on 7-point Likert scales (1 =  “not at all”; 7 =  “very much”) for each artwork. Besides its relation to the theme of the exhibition, we asked for ambiguity because it is an important feature of art experience, especially of modern and contemporary art [35]–[39]. The order of rating scales for each artwork and the order of artworks were randomized in both groups.

At the end of each session, participants filled out two questionnaires on their art and photography expertise. Similar to Belke, Leder, and Augustin [40], the questionnaires contained items measuring participant's general interest in and explicit knowledge about art or photography, respectively.

## Results

Confirming their layperson status, participants scored low on expertise in art (*M* = 33.34 on a maximum score of 100) and photography (*M* = 34.52 on a maximum score of 100). Importantly, groups did not differ significantly in their average level of interest in and knowledge about art or photography, respectively (art expertise: *M*_MG_ = 35.32, *M*_LG_ = 31.36, *t*(42)  = −1.21, *p* = .233; photography expertise: *M*_MG_ = 36.45; *M*_LG_ = 32.64, *t*(42) = −1.66, *p* = .103).

### Viewing time

**Figure 1:**
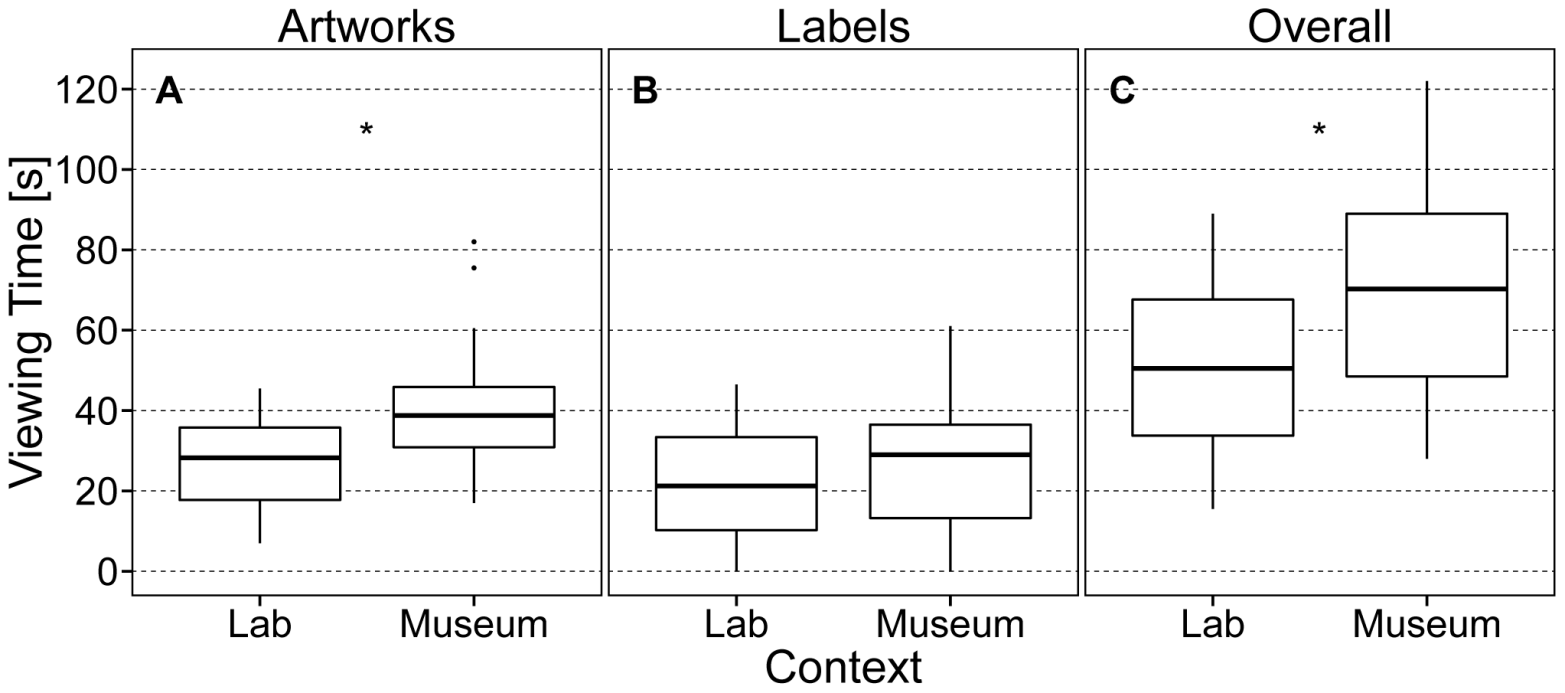
Differences in viewing time between contexts. Boxplots for A) viewing time for artworks (VT-A), B) viewing time for labels (VT-L), and C) overall viewing time (VT-O = VT-A+VT-L) split by context. Asterisks indicate significant differences between medians (*p*<.05). Lab  =  Laboratory.

Viewing time for each artwork and label was analyzed with SMI Begaze 3.2 software. By defining each artwork and label as region of interest (ROI) and computing the sum of all fixation and saccade durations for each ROI, we obtained viewing time measures separately for each artwork and label for each participant. Next, we derived three measures of viewing time: viewing time for artworks (VT-A), viewing time for labels (VT-L), and overall viewing time (VT-O = VT-A+VT-L). Figure 1 shows all viewing time measures for each context. Because viewing time measures were positively skewed, non-parametric statistical tests were used for analysis. Time spent reading the labels (*Mdn_MG_* = 29.00 s, *Mdn_LG_* = 21.25 s) did not significantly differ between contexts (*p* = .391). However, viewing time for artworks (*W* = 108.5, *p* = .002, r = −0.47) and overall viewing time (*W* = 155, *p* = .042, r = −0.31) differed significantly between contexts. Compared to the laboratory context (*Mdn* = 28.25 s), people in the museum spent more time looking at artworks (*Mdn* = 38.75 s). Consequently, overall viewing time was also longer in the museum (*Mdn* = 70.25 s) than in the laboratory context (*Mdn* = 50.50 s).

### The experience of art

**Figure 2:**
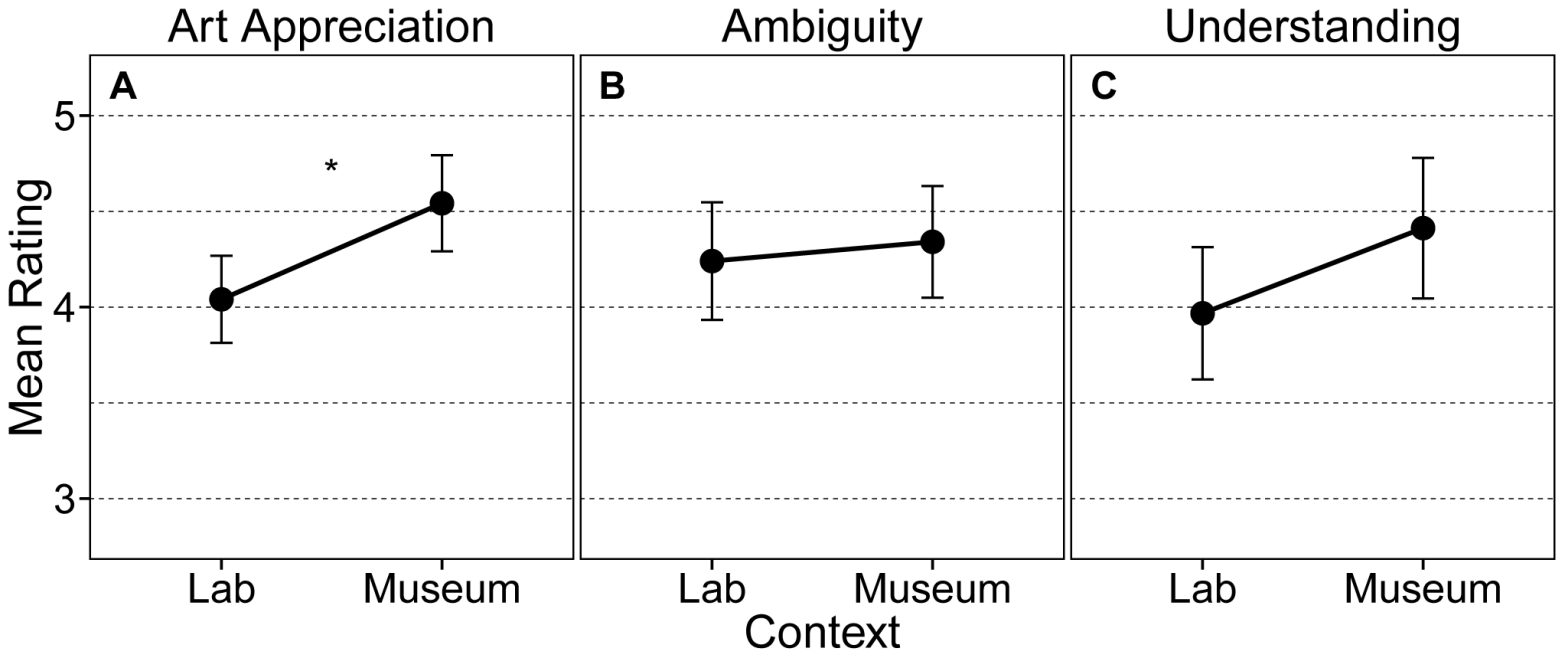
Differences in art appreciation, ambiguity, and understanding between contexts. Mean ratings for A) art appreciation, B) ambiguity, and C) understanding split by context. Asterisks indicate significant differences (*p*<.05). Lab  =  Laboratory. Error bars represent 95% CI.

Exploring the relationship among the mean liking, interest, understanding, and ambiguity ratings, we found that liking and interest were highly correlated (*r* = .85). Therefore, we decided to aggregate these two variables and form a new one, called appreciation, by computing the mean of liking and interest [25]. Figure 2 depicts mean ratings for appreciation, understanding, and ambiguity for each context. In order to ascertain whether there was an enhanced art experience in the museum, we performed a multivariate analysis of variance (MANOVA) with appreciation, understanding, and ambiguity ratings as dependent variables and context (museum vs. laboratory) as independent variable. This analysis revealed a significant effect of context, *F*(3, 40)  = 3.52, *p* = .024. To further explore this effect, separate ANOVAs were conducted for each rating scale. In line with our hypothesis, appreciation was enhanced in the museum compared to the laboratory context, *F*(1,42)  = 9.50, *p* = .004, meaning that participants liked the artworks more and found them more interesting in the museum than in the laboratory. Additionally, there was also a trend towards significance for a higher sense of understanding in the museum than compared to the laboratory context, *F*(1,42)  = 3.37, *p* = .073. However, we found no difference between contexts with regards to the evaluation of ambiguity (*p* = .623).

### Relation between viewing time and the experience of art

To ascertain the factors that influence how much time people spend looking at art and reading their labels, we used linear mixed effects modeling to predict viewing time for artworks and labels from all art experience ratings (appreciation, understanding, and ambiguity) and the context in which artworks were seen. Additionally, due to the effect of physical stimulus properties on viewing time [11], [41], we also added artwork size and number of words on the label to the statistical models. Moreover, we also considered the order of artworks because of the *museum fatigue* phenomena [20].

For the mixed effects model analyses, we used R version 2.15.1 [42] and the R package “lme4” [43]. In contrast to conventional multiple regression analysis, mixed effects modeling allowed us to examine group differences in within-person relationships without aggregating over artworks or subjects. According to our data structure, artworks and participants were fully crossed random effects – each artwork was viewed by each participant [44], [45]. Viewing time for artworks and labels were outcome variables in two separate models.

#### Predicting viewing time for artworks

The first model aimed to predict viewing time for artworks. Here, appreciation (compounding liking and interest), understanding, ambiguity, and context were the main predictors of interest. Additionally, we also included artwork size and the order of artworks in the model to statistically control for their influence on viewing time for artworks. Each predictor (except for context) was grand mean centered for a meaningful interpretation of the model's intercept and to reduce multicollinearity. Due to a positively skewed distribution, the outcome variable (viewing time for artworks) was natural log-transformed to approximate normal distribution of the residuals. In the first step, we assessed whether applying a mixed effects model approach was justified. Therefore, we used a likelihood ratio test to compare a fixed intercept model (null model) with a model including random intercepts for artworks and participants (no predictors in either model). The random intercept model showed a significantly better fit than the null model, χ^2^(2)  = 312.65, *p*<.001. This means that allowing intercepts to vary among artworks and participants reduced the unexplained variance and therefore improved the model's fit. As a second step, each predictor and each interaction between predictors and context was added to the model. An F-test with Kenward-Roger approximation (using the KRmodcomp function in the pbkrtest package [46]) revealed that adding these fixed effects significantly improved the model's fit, *F*(11,134.41)  = 8.56, *p*<.001. In the final step, each non-significant main effect or interaction was excluded from the model if it did not worsen the overall model's fit. This was done with an automatic backward selection using the *step* function from the “lmerTest” package [47].

**Figure 3:**
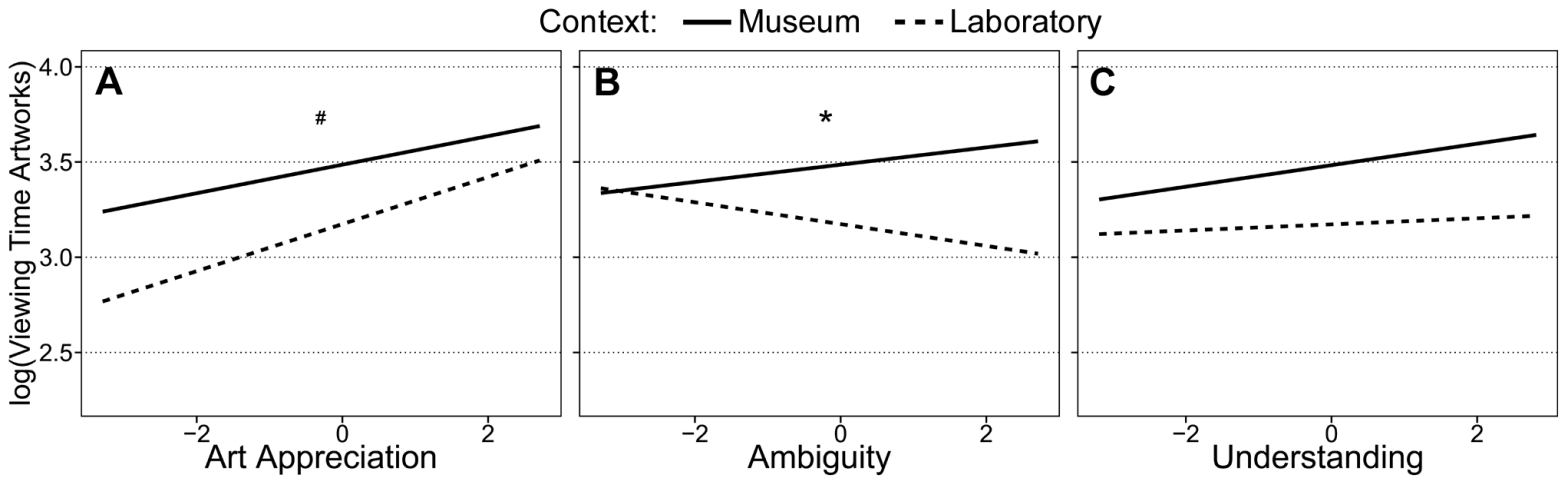
Relationships between viewing time for artworks and art experiences by context. Relationships according to mixed effects model analysis between log transformed viewing time for artworks and A) art appreciation, B) ambiguity, and C) understanding ratings, respectively. Asterisks indicate significant (*p*<.05) and hashes indicate trends (*p*<.10) for different slopes for the museum (solid) and laboratory (dashed) context.

The final model revealed a main effect of artwork order, *b* = −0.089, *SE* = 0.028, *t*(533)  = −3.09, *p* = .009. Thus, independently of context, viewing time decreased from the first to the last artwork of the exhibition (while holding all other predictors constant at grand mean level). The influence of the second control variable, artwork size, was not significant and, therefore, was excluded from the final model. This was probably because allowing the intercepts to vary among artworks already accounted for the variance in viewing time for artworks due to artwork size. Each of the main predictors—appreciation, understanding, ambiguity, and context—significantly predicted viewing time for artworks (Figure 3). Understanding was positively related with viewing time (*b* = 0.035, *SE* = 0.016, *t*(533)  = 2.15, *p* = .032). Thus, in both contexts, higher sense of understanding was associated with longer viewing time. In accordance with the viewing time analysis reported above, we found a main effect of context (*b* = −0.312, *SE* = .131, *t*(42)  = −2.37, *p* = .022; with museum as reference group). This indicates a significant difference between contexts in viewing time for artworks. Interestingly, the analysis also revealed interactions between appreciation and context as well as ambiguity and context. People spent more time looking at artworks the more they appreciated them in the museum (*b* = 0.075, *SE* = 0.021, *t*(533)  = 3.46, *p*<.001) and in the laboratory context (*b* = 0.123, *SE* = 0.019, *t*(533)  = 6.17, *p*<.001). However, appreciation predicted viewing time better in the laboratory than compared to the museum context (*b* = 0.048, *SE* = 0.024, *t*(533)  = 1.94, *p* = .052; for the interaction between appreciation and context with museum as reference group). The impact of ambiguity on viewing time also differed significantly between contexts (*b* = −0.102, *SE* = 0.029, *t*(533)  = 3.43, *p* = .001; for the interaction between ambiguity and context with museum as reference group). While in the museum context more ambiguous artworks received longer looks (*b* = 0.045, *SE* = 0.022, *t*(533)  = 1.99, *p* = .046), the opposite effect occurred in the laboratory context, where more ambiguous artworks received shorter looks (*b* = −0.057, *SE* = 0.021, *t*(533)  = −2.74, *p* = .006).

#### Predicting viewing time for artwork labels

Building up the second mixed effects model was similar to the first, except that now viewing time for artwork labels was the outcome variable. Instead of artwork size, we considered the number of words on the label as a control variable. Because some participants did not read all labels, the viewing time data contained zeros. Therefore, in contrast to the analysis of viewing time for artworks, we used log(x+1) transformation to approximate normality of residual viewing time for artwork labels. The analysis process was the same as described above for the previous analysis. Compared to the null model, allowing the intercepts to vary among participants and artworks significantly improved the model's fit, χ^2^(2)  = 62.03, *p*<.001. Adding all single predictors and its interactions with context significantly reduced the unexplained variance, *F*(7,68.06)  = 11.934, *p*<.001. Again, the final model was obtained after eliminating all non-significant predictors and interactions without decreasing the model's fit.

**Figure 4:**
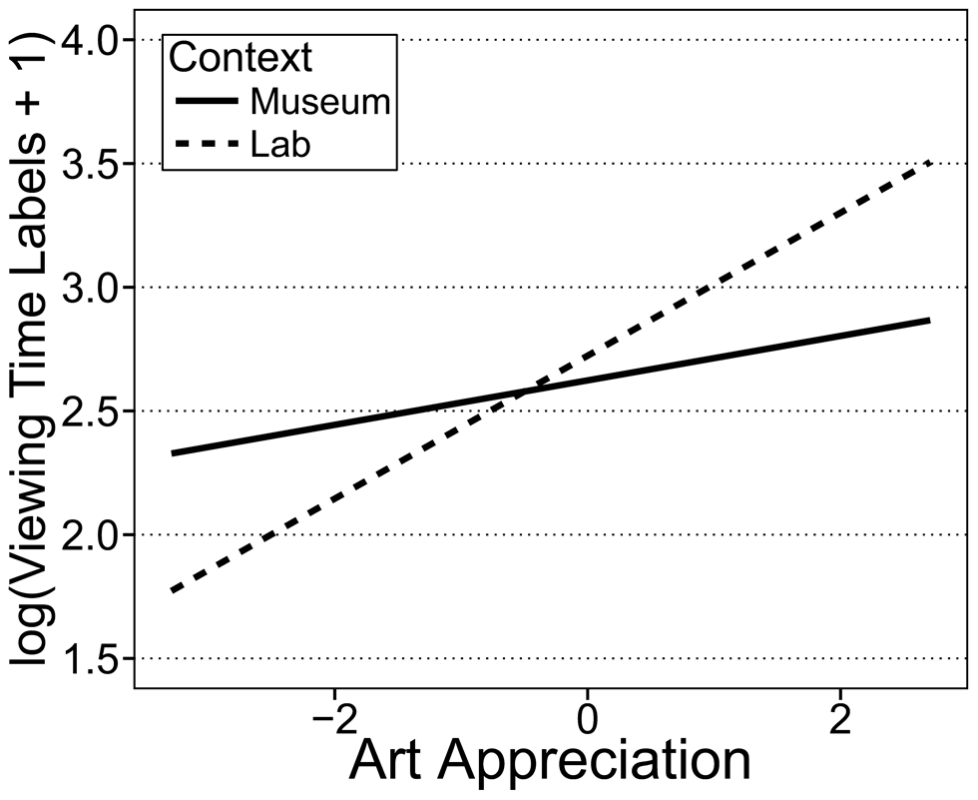
Relationship between viewing time for labels and art appreciation by context. Relationship according to mixed effects model analysis between log(x+1) transformed viewing time for labels and art appreciation ratings. Lab  =  Laboratory. Slopes for the museum (solid) and laboratory (dashed) context are significantly different (*p*<.05).

In the final model, viewing time for artwork labels was predicted by appreciation, the number of words on the labels, and the order of artworks. The effect of all of these predictors, however, differed between contexts. In both contexts, people spent more time reading labels for artworks they appreciated more (museum: *b* = 0.089, *SE* = 0.043, *t*(533)  = 2.10, *p* = .036; laboratory: *b* = 0.288, *SE* = 0.037, *t*(533)  = 7.71, *p*<.001; Figure 4). Similarly to viewing time for artworks, this impact of appreciation on viewing time for labels was stronger in the laboratory than in the museum context (*b* = 0.199, *SE* = 0.054, *t*(533)  = 3.66, *p*<.001; for the interaction between appreciation and viewing time for labels with museum context as reference group). Other factors such as the number of words on the label (*b* = 0.005, *SE* = 0.002, *t*(533)  = 2.49, *p* = .024) or the order of artworks (*b* = −0.089, *SE* = 0.026, *t*(533)  = −3.39, *p* = .003) were only significant in the museum but not in the laboratory context (*p*s>.17).

## Discussion

The goal of this study was to ascertain how context affects viewing time, the experience of art, and the relationship between viewing time and the experience of art. In line with our hypotheses, (i) participants viewing the artworks in the museum liked them more and found them more interesting than participants viewing them in the laboratory. Compared to the laboratory, (ii) average viewing time for artworks (but not for labels) was longer in the museum context. Associating viewing time and experience showed that (iii) participants' experience predicted viewing time for artworks and labels. Interestingly, the impact of certain predictive factors on viewing time was modulated by context. We discuss each finding in more detail below.

### Differences in viewing time between contexts

We expected the museum context to foster a prolonged and focused reception of art, indicated by longer viewing times. Our data supports this assumption: Whereas the groups spent similar amounts of time reading labels, on average participants in the museum looked longer at artworks than people in the laboratory (In the museum: *Mdn* = 38.75 s; In the laboratory: *Mdn* = 28.25 s). Compared to other studies measuring the time museum visitors spend viewing artworks (e.g. [21], median overall viewing time: 17 s), participants in the current study in general, and the museum group in particular, spent considerably more time on looking at artworks. The differences between the methods employed for data collection as well as the overall number of exhibited artworks might explain the differences in average viewing time between studies. In contrast to Smith and Smith's [21] unobtrusive observational method, our participants knew that their eye movements were being recorded. Thus, a social desirability bias on viewing time cannot be totally excluded, although we clearly emphasized that participants could look at the artworks for as long as they wanted. Another explanation refers to the relation between viewing time and the number of exhibited artworks. Melton [19] already showed that the higher the number of artworks in an exhibition, the shorter the time spent viewing each of them. Accordingly, considering that Smith and Smith's participants were spontaneous visitors of the Metropolitan Museum of Art in New York, the large number of exhibited artworks probably reduced average viewing time.

### Differences in the experience of art between contexts

The appreciation of art differs between the museum and the laboratory context. In our study, artworks were liked more and found more interesting in the museum context. On the other hand, the evaluation of ambiguity did not differ between contexts and the sense of understanding only tended to be higher in the museum. This suggests that the museum context enhances especially those aspects of art experience that are more closely linked with emotion (liking and interest). Although most studies that investigated emotional experiences during art perception were actually conducted in the laboratory context [48]–[51], our findings indicate that emotional responses to art may be stronger in more usual settings.

Our results are well in line with previous findings, which reported higher interestingness and pleasantness ratings for original artworks in the museum compared to digital reproductions presented on a laboratory computer screen [33]. Nadal, Brieber, and Leder [34] obtained similar results using a within-subject design and also found that artworks in the museum were liked more, found more interesting and experienced as more positive and arousing. Thus, although these studies were conducted in several museums using different artworks and different experimental designs (between- and within-subject), they all support the notion that there is an enhanced art experience in the museum.

Among several factors that might contribute to this enhanced art experience in the museum, people's aesthetic attitude while beholding an artwork and the authenticity of the artwork itself might play a decisive role. The museum context enhances the artistic status of the exhibited objects [17] and, consequently, facilitates the adoption of an aesthetic orientation associated with increased affective processing [52]. In the museum, visitors with an aesthetic orientation encounter genuine artworks. Authenticity is an important factor increasing the monetary value [28] and aesthetic evaluation of art [30]. It remains unclear, however, whether the physical context, the authenticity of the artwork, or an interaction between them determines the enhanced art experience in the museum. Therefore, further studies are required to disentangle the effects of physical context and authenticity on art experience.

### Context modulates the relation between art experience and viewing time

Our analyses demonstrate that there is a relationship between people's experience of art and how much time they choose to view art and reading the labels. Furthermore, the relationship between viewing time and art experience was modulated by context. These context effects show that, over and above quantitative differences in viewing time and art experience between contexts, effects identified under laboratory conditions are not necessarily transferable to other settings. Additionally, building up on research which found that art-specific information affects appreciation [23]–[25], our analysis of predicting viewing time for labels offers an explanation which factors actually drive information seeking. Hereafter we discuss how the predictors of main interest—appreciation, ambiguity, and understanding—affect viewing time for artworks and labels.

In both contexts, viewing time for artworks and labels increased with appreciation (liking and interest). The strength of this relationship, however, tended to vary with context. People's appreciation showed a slightly stronger relationship with viewing time for artworks and labels in the laboratory than in the museum context. Thus, appreciation based on liking and interestingness seems to be a more important predictor of viewing time in the laboratory than in the museum context. This suggests that longer viewing times (for artworks) in the museum context cannot be explained by an enhanced appreciation alone. Additional factors such ambiguity need to be considered.

It has been argued that ambiguity is an important aspect of aesthetic experience [35]. Our results showed, however, that the effect of ambiguity on time spent viewing artworks substantially differed between contexts. While there was a positive relation between ambiguity and viewing time in the museum, participants in the laboratory showed a negative relationship. This is particularly interesting in relation to the finding that participants in the museum and the laboratory did not differ in how they evaluated ambiguity. Thus, although people's experience of ambiguity might be similar in different contexts, its impact on people's viewing behavior differs. The museum context promotes that people engage with and delve into the ambiguity in artworks and invest more time on resolving it, while in the laboratory context, the point in time where people decide to refocus their attention on another artwork comes sooner for the more ambiguous artworks. Therefore, the museum context changes how ambiguity affects people's exploration behavior.

Similar to the effects of appreciation, understanding also significantly predicted viewing time for artworks in both contexts. Increased understanding was related to an increase in viewing time for artworks. However, the subjective impression of understanding the artist's work was not related to the time people spent on reading the labels. This is surprising because text information provided on labels is supposed to assist the beholder in understanding the technique, theme, and the historical, autobiographical, or sociopolitical context. Especially with respect to contemporary art, the “need for commentary” should be very strong [53]. Therefore, in accordance with previous research on the effect of information on understanding [23], [25], we expected a positive relationship between understanding and viewing time for labels. One possible explanation for our finding could be that the labels in this exhibition were not informative enough to affect understanding and, thus, people did not benefit from reading the text information. The lack of the relation between understanding and viewing time for labels, however, remains unclear and needs further studies.

Our general finding that art experience is positively related to viewing time differs from Heidenreich and Turano's [22] who did not find any significant correlations. Our analysis suggests that art appreciation is less strongly related to viewing time in the museum than in laboratory context. Given that Heidenreich and Turano collected their data in the museum with a small sample of only four participants, it is likely that these differing findings occur because their analysis was underpowered.

### Limitations

Similar to most studies in the field of empirical aesthetics, the current study only recruited (psychology) students whose intrinsic motivation to see the artworks is uncertain. This approach guarantees homogeneous samples with regards to factors such as age, socioeconomic status, and education and facilitates the control of the level of art expertise. Nevertheless, further studies should be conducted with spontaneous art museum visitors [21], [54], [55] to raise the level of ecological validity and enlarge our knowledge about art experience in the museum.

## Conclusion

Empirical aesthetics has mostly focused on how stimulus or personal factors contribute to the appreciation of art. Our results demonstrated that context affects the experience of art, the amount of time people spend on art, and, in turn, that viewing time is related to art experience. Therefore, context needs to be considered as an important modulating factor in the appreciation of art. Our results suggest that time and context constitute more than framing dimensions for the experience of art. Owing to the impact of the temporal unfolding of cognitive and affective processes on the appreciation of art, and to their contextual sensitivity, time and context can be regarded as fundamental elements in the elaboration of the experience of art. Nevertheless, more comparative studies between art perception in the laboratory and the museum are required to fully ascertain which processes, experiences, or behaviors are transferable from one context to the other. In light of the finding that art experience is enhanced in the museum, future research should focus on specific factors that lead to this art museum experience.
